# From seaside to bedside: Current evidence and future perspectives in the treatment of breast cancer using marine compounds

**DOI:** 10.3389/fphar.2022.909566

**Published:** 2022-09-08

**Authors:** Rita De Sanctis, Flavia Jacobs, Chiara Benvenuti, Mariangela Gaudio, Raul Franceschini, Richard Tancredi, Paolo Pedrazzoli, Armando Santoro, Alberto Zambelli

**Affiliations:** ^1^ Department of Biomedical Sciences, Humanitas University, Pieve Emanuele, Milan, Italy; ^2^ Medical Oncology and Hematology Unit, Humanitas Cancer Center, IRCCS Humanitas Research Hospital, Rozzano, Milan, Italy; ^3^ Department of Chemistry, Università degli studi di Milano Statale, Milan, Italy; ^4^ Medical Oncology Unit, ASST Melegnano Martesana, Ospedale A. Uboldo, Milan, Italy; ^5^ Department of Internal Medicine and Medical Therapy, University of Pavia, Pavia, Italy; ^6^ Medical Oncology Unit, Fondazione IRCCS Policlinico San Matteo, Pavia, Italy

**Keywords:** breast cancer, eribulin, trabectedin, lurbinectedin, antibody drug conjugate (ADC), cytarabine, plocabulin

## Abstract

To date, only few marine natural compounds have been proved to be active in breast cancer (BC). The main marine-derived drugs that have been studied for the treatment of BC are tubulin-binding agents (eribulin and plocabulin), DNA-targeting agents (cytarabine and minor groove binders—trabectedin and lurbinectedin) and Antibody-Drug Conjugates (ADCs). Notably, eribulin is the only approved cytotoxic drug for the treatment of advanced BC (ABC), while cytarabine has a limited indication in case of leptomeningeal diffusion of the disease. Also plocabulin showed limited activity in ABC but further research is needed to define its ultimate potential role. The available clinical data for both trabectedin and lurbinectedin are of particular interest in the treatment of BRCA-mutated tumours and HR deficient disease, probably due to a possible immune-mediated mechanism of action. One of the most innovative therapeutic options for the treatment of BC, particularly in TNBC and HER2-positive BC, are ADCs. Some of the ADCs were developed using a specific marine-derived cytotoxic molecule as payload called auristatin. Among these, clinical data are available on ladiratuzumab vedotin and glembatumumab vedotin in TNBC, and on disitamab vedotin and ALT-P7 in HER2-positive patients. A deeper knowledge of the mechanism of action and of the potential predictive factors for response to marine-derived drugs is important for their rational and effective use, alone or in combination. In this narrative review, we discuss the role of marine-derived drugs for the treatment of BC, although most of them are not approved, and the opportunities that could arise from the potential treasure trove of the sea for novel BC therapeutics.

## 1 Introduction

More than 50 years ago, the very first drug extracted from the sea, cytarabine, arrived in clinics. Cytarabine (also known as Ara-C, Cytosar-U^®^) was isolated from a marine sponge and demonstrated activity against cancer cells *via* blocking DNA polymerase (Dyshlovoy and Honecker, 2018). Due to its biological activity, the drug was approved by the Food and Drug Administration (FDA) for the treatment of leukaemia in 1969, and since then Cytarabine has remained a relevant player in the therapeutic strategy for haematological malignancies.

Inexplicably, shortly after this initial success, the history of marine-drug development suffered a prolonged setback and appeared to have ended. In fact, no further compounds were approved by health authorities for almost 40 years and the search for novel anticancer drugs from natural sources had also declined in favour of computational and high-throughput screening approaches to rational drug design.

At the beginning of the 21st century, however, the development of medicines from the sea experienced a renaissance and gained a new momentum (Stonik, 2009; Brönstrup and Sasse, 2020). Supported by modern biochemical approaches, the renewed interest in marine anticancer derivatives has led to the identification of novel marine molecules that were undetectable in the past and have a specific mechanisms of action.

The role of marine natural products as candidates anticancer drugs is now widely recognised and represents an important field of research and development. In the last two decades, the number of the available marine-drugs has almost doubled (Dyshlovoy and Honecker, 2015) and as of March 2022, the list of the marine-derived drugs officially approved by the regulatory agencies for cancer treatment encompasses 12 compounds, with Eribulin the only one approved for breast cancer (BC), including in a chronological list: 1) the spongian nucleoside Cytarabine, 2) the spongian macrolide Eribulin mesylate, 3) the Brentuximab vedotin, 4) the ascidian alkaloid Trabectedin, 5) the marine-derived HDAC inhibitor Panobinostat, 6) the ascidian depsipeptide Plitidepsin, 7) the Polatuzumab vedotin, 8) the Enfortumab Vedotin, 9) the ascidian alkaloid-derived Lurbinectedin, 10) the Belantamab Mafodotin, 11) the Disitamab Vedotin, and 12) the Tisotumab vedotin.

Besides, more than 30 additional candidate anticancer marine-derived molecules are currently in various stages of development (Ha et al., 2019), most of which are antibody-drug conjugates (ADC) and some of which have a promising activity against BC.

**FIGURE 1:**
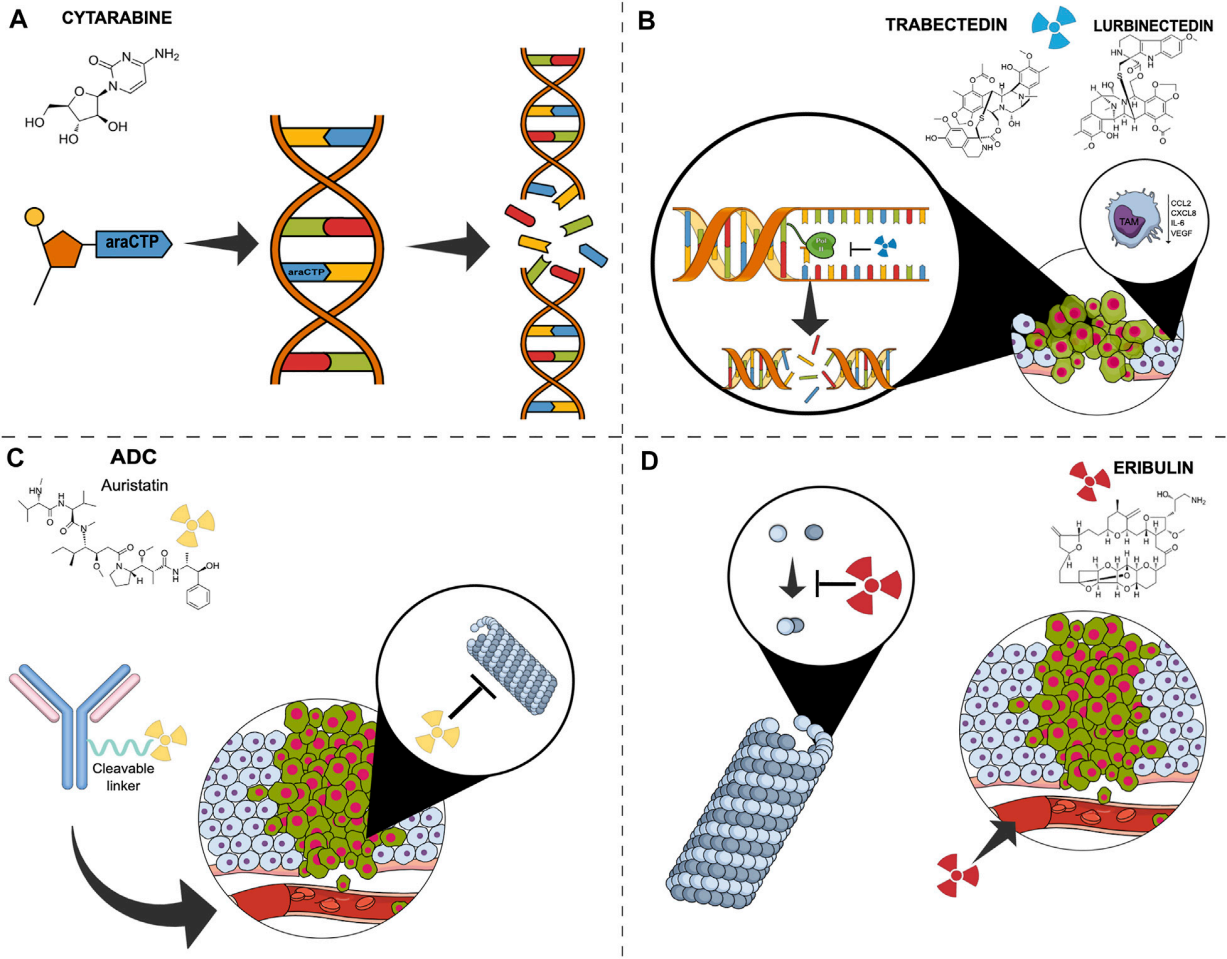
Simplified mechanisms of action of marine derivate compound in breast cancer. **(A)** Cytarabine is incorporated into DNA as its activated form, ara-cytidine 5′-triphosphate, and promotes abnormal fragment binding of newly synthesised DNA, leading to apoptosis. **(B)** Trabectedin and Lurbinectedin bind to the minor groove of DNA inhibiting transcription, resulting in double-strand DNA breaks and cell death. They also affect the tumour microenvironment by negatively modulating tumour-associated macrophages’ activity. **(C)** Antibody-drug conjugates consist of a monoclonal antibody bound to a payload, generally monomethylauristatin E, a cytotoxic compound that binds to tubulin, leading to both disruption of mitotic spindle assembly and arrest of tumour cells in the mitotic phase of the cell cycle. **(D)** Eribulin inhibits the microtubule growth phase forming non-productive tubulin aggregates. It also acts on angiogenesis. Abbreviations: ADC, antibody drug conjugate; ara-CTP, ara-Cytidine-5′-triphosphate; CCL2, C-C Motif Chemokine Ligand 2; CXCL8, C-X-C Motif Chemokine Ligand 8; DAR, Drug-antibody Ratio; EGFR2, epidermal growth factor receptor; IL6, Interleukin 6; mgDNA, minor groove DNA; MMAE, monomethylauristatin E; RNApol, RNA polymerase; TAM, tumor-associated macrophages; tc-NER, Transcription-coupled nucleotide excision repair; VEGF, Vascular-Endothelial Growth Factor.

In this narrative review, we discuss the potential role of marine-derived drugs in the treatment of BC (Figure 1) and the opportunities offered by the potential treasure trove of the sea for novel BC therapeutics.

## 2 Tubulin-binding agents

**FIGURE 2:**
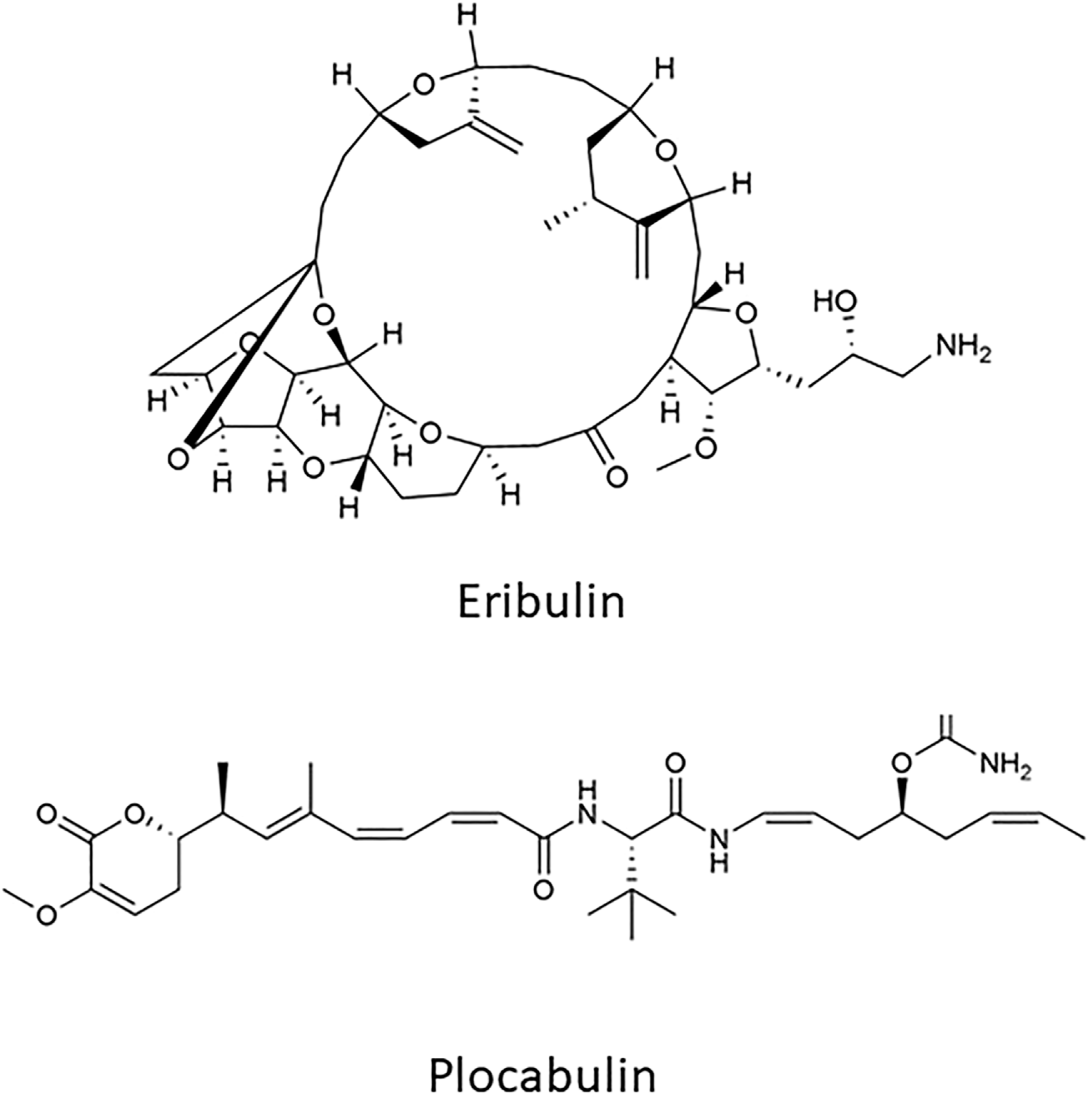
Chemical structure of Eribulin and Plocabulin.

Eribulin mesylate and Plocabulin are two of the major anti-microtubule cytotoxic agents isolated from marine sources (Figure 2).

### 2.1 Eribulin

Eribulin mesylate is one of the most important marine compounds studied for its anticancer activity. It was isolated for the first time in 1986 from a natural product, the black sea sponge off the coast of Japan, *Halichondria okadai*. Despite its interesting mechanism of action with promising antitumor effects, its complex structure and the presence of contaminants made it difficult to use until 1992 when the total synthesis of halicondrin B was completed and many synthetic analogues were successfully developed, including eribulin.

Eribulin has a unique mechanism of action: unlike other compounds such as taxanes and vinca alkaloids, it exerts its cytotoxic effect by suppressing microtubules polymerization without affecting depolymerization, thereby preventing spindle formation, which ultimately leads to mitotic arrest and subsequent cell apoptosis (Gourmelon et al., 2011). Because of this mechanism of action, eribulin may show activity also in case of taxane-resistant tumour cell lines.

Moreover, some preclinical studies of human BC models have suggested that eribulin may also have a non-mitotic activity. Indeed, eribulin can affect tumour microenvironment and restore its vasculature and perfusion, downregulating the expression of vascular endothelial growth factor (VEGF) and TGFbeta genes. These alterations could be responsible for the potential enhancement of the subsequently administered chemotherapy by both reducing hypoxia-driven chemoresistance and increasing the intratumoral delivery of the drug. As a consequence, eribulin might contribute to modify the advanced BC (ABC) disease-trajectory and the post-progression survival outcome, as observed in phase III trials. Besides, preclinical studies have also shown that eribulin can affect the epithelial-mesenchymal transition process (EMT) (Ueda et al., 2016) and can favourably impact on immunological tumour microenvironment (De Sanctis et al., 2018). Notably, in triple-negative BC (TNBC) patients, the high level of tumour-infiltrating lymphocytes (TILs) has been shown to predict the efficacy of eribulin, with a significant DFS improvement (Kashiwagi et al., 2017), supporting a drug-related synergistic engagement of the anticancer immune-response. Following these suggestions, eribulin was tested in association with PD-1 inhibitors. Interesting preliminary results were reported in the phase Ib/II ENHANCE1 study, which evaluated the combination of eribulin with pembrolizumab in PD-L1-positive advanced TNBC patients. An ORR of 26% was observed, which was higher than the ORR observed in the past with either eribulin or pembrolizumab as monotherapy, in the same setting of patients (ORR 10 and 21%, respectively) (Tolaney et al., 2021).

Eribulin was approved by the regulatory agencies in the United States (November 2010) and in Europe (March 2011) for the treatment of ABC patients who had received at least one or two lines of prior chemotherapy, respectively. In Europe, the recommended dose of eribulin refers to the active substance (eribulin, 1.23 mg/m2) whereas in the United States to the salt form (eribulin mesylate, 1.4 mg/m2) and it should be administered intravenously over 2–5 min on Days 1 and 8 of every 21-days cycle (Goel et al., 2009).

The eribulin approval derived from the results of the EMBRACE trial, a randomized, open-label, phase III study, which demonstrated for the first-time ever in heavily pre-treated ABC the benefit of a cytotoxic single-agent in terms of statistically significant overall survival (OS) improvement, compared to the best treatment physician’s choice (TPC) (13.1 vs. 10.6 months, *p* = 0.041) (Cortes et al., 2011). In a subsequent phase III trial (study E-301), pre-treated ABC patients were randomized to receive eribulin or capecitabine as first, second or third-line therapy (Kaufman et al., 2015). Eribulin failed to demonstrate superiority over capecitabine, showing similar results in terms of OS (15.9 vs. 14.5) and no differences in progression free survival (PFS) and ORR. Notwithstanding this finding, however, a subsequent post-hoc pooled analysis of the EMBRACE and E-301 trials showed that eribulin prolonged OS in the entire patient population and in all patient subgroups (Twelves et al., 2014). Although real-world evidence supports the efficacy of eribulin in chemo-pretreated ABC regardless of cancer subtypes (Pedersini et al., 2018), a greater clinical benefit was observed in the case of TNBC (Twelves et al., 2014).

Besides TNBC, emerging data suggest that eribulin is also effective and tolerable in patients with HER2-positive ABC. Given the promising results of clinical trials with anti-HER2 agents in combination with conventional chemotherapies, the use of eribulin with trastuzumab was investigated in several studies. This combination was tested in a phase II trial for the first-line treatment of HE2-positive ABC and showed an ORR of 71.2% with a median PFS of 11.6 months (Sakaguchi et al., 2018). Another phase II trial assessed the combination of eribulin with the dual antiHER2 block, trastuzumab and pertuzumab, in taxane-pretreated HER2-positive ABC, showing favourable outcome in terms of ORR and prolonged PFS (Araki et al., 2017).

There is only little evidence regarding the role of eribulin in early BC. In the neoadjuvant setting, a phase II trial evaluated for the first time the combination of eribulin with carboplatin in TNBC, reporting an encouraging 43% of pathologic complete response (Kaklamani et al., 2015).

Similarly, in the adjuvant setting, a single pilot experience evaluated the feasibility of the combination of eribulin with capecitabine (days 1–14 of a 21-days cycle) in HR-positive HER2-negative, stage I–II early BC, with preliminary interesting results (Smith et al., 2016).

The most frequent adverse events (AEs) associated with eribulin were neutropenia, fatigue and neuropathy. As reported in the EMBRACE study, neutropenia occurred in 22%–49% of patients and it was easily managed with dose delay, dose reductions or administration of stimulating growth factors. Neuropathy was the most common non-hematologic AEs leading to a limited treatment discontinuation in EMBRACE trial [24 (5%) of 503 patients] (Cortes et al., 2011). Alternative schedules of administration (e.g., biweekly) are being evaluated to derive a better toxicity profile and treatment tolerance.

### 2.2 Plocabulin

Plocabulin is a novel tubulin-binding agent, isolated for the first time from the Madagascan sponge *Lithoplocamia lithistoides*, currently produced by total synthesis (Pera et al., 2013; Martínez-Díez et al., 2014). Unlike eribulin, plocabulin binds with high affinity to a new site in the β-tubulin plus end, inhibiting the microtubule growing at a very low concentration. The resulting microtubules instability affects the cellular cycle both during interphase and mitosis, leading to alteration in cell shape, trafficking, signalling, transportation, migration and, ultimately, cell apoptosis.

Moreover, the inhibition of microtubule dynamics in endothelial cells leads to alterations in tumour vascular architecture. These antiangiogenic effects, obtained with a lower dose than the cytotoxic one, contribute to enhancing plocabulin’s anticancer activity (Galmarini et al., 2018). Worthy of note, plocabulin preserved its effect even in cells expressing the P-gp multidrug efflux pump, typically resistant to vinorelbine and paclitaxel, two well-known and extensively used drugs in BC (Martínez-Díez et al., 2014). Both *in vitro* and *in vivo* studies, Plocabulin exhibits a promising cytotoxic effect on breast tumour cells (Pera et al., 2013).

The first-in-human phase I trial (NCT01299636) of plocabulin in patients with several advanced solid tumour included five patients with ABC. Of them, three achieved stable disease (SD) as the best response, with maximum tumour shrinkage of 28%. The single BC patient who derived the greatest benefit was heavily pretreated (10 prior lines) and reached an interesting PFS of 6 months.

These preliminary signals of anticancer activity came with some toxicities, being the peripheral sensory neuropathy the most common and severe AEs, especially in patients who already had the chemotherapy-induced peripheral neuropathy (CIPN) at baseline (i.e., oxaliplatin). Other common plocabulin-related AEs were mild or moderate, including fatigue, nausea, alopecia, vomiting and abdominal pain. The main severe haematological toxicity was anaemia (23% of grade 3), being neutropenia and thrombocytopenia mild or moderate. Most biochemical abnormalities were grade 1 or 2 and included alanine aminotransferase (ALT) and aspartate aminotransferase (AST) increase and hypoalbuminemia (Elez et al., 2019).

Another prospective phase I trial (NCT02533674) testing plocabulin in combination with gemcitabine in selected advanced solid tumours, including 4 ABC, has been completed, but data on drug efficacy have not yet been reported.

Unfortunately, despite the interesting rationale for the potential role of plocabulin in tumours resistant to other antimicrotubular agents, its activity in advanced BC has not been further investigated in other trials.

## 3 DNA-targeting agents

### 3.1 Cytarabine

**FIGURE 3:**
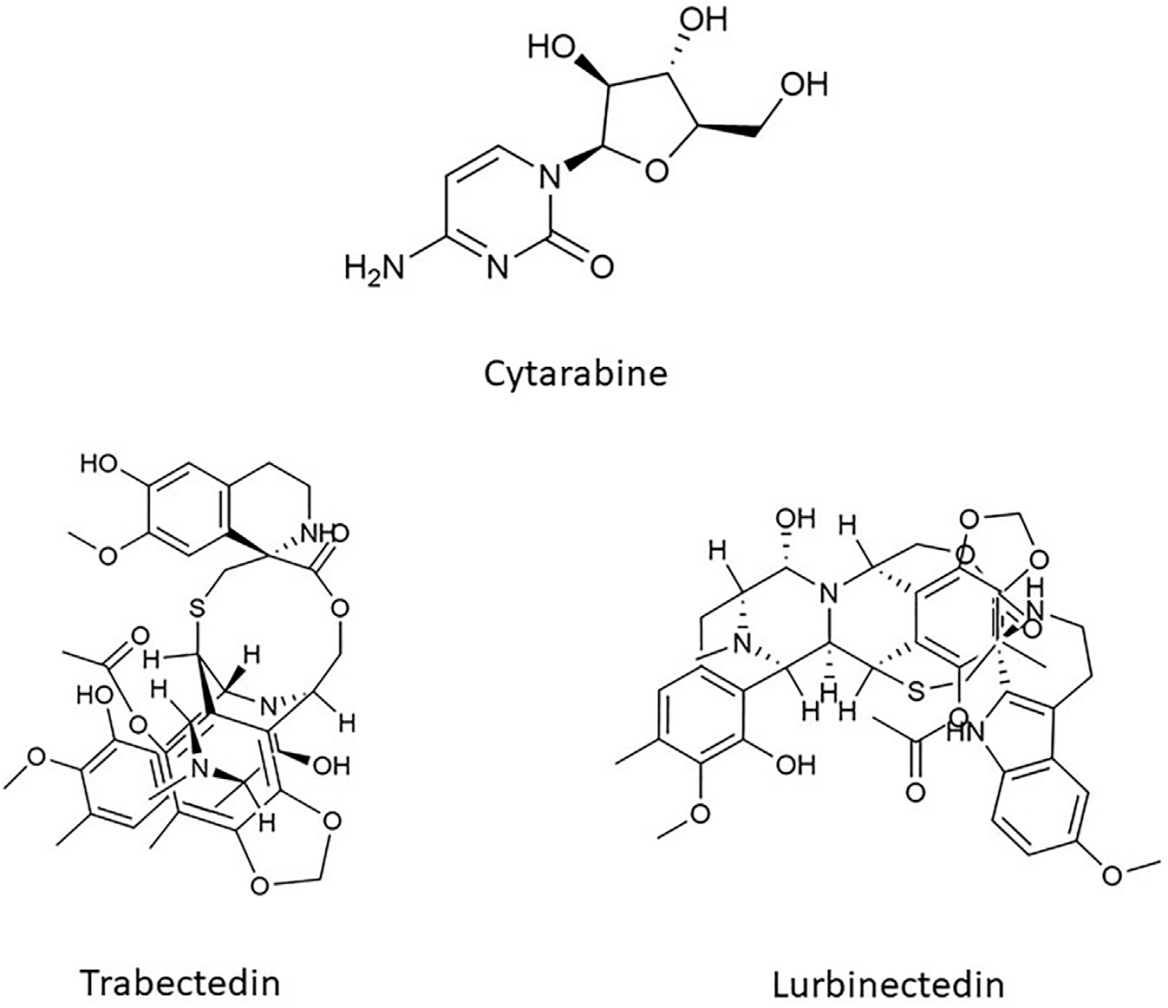
Chemical structure of Cytarabine, Trabectedin, and Lurbinectedin.

Cytarabine, also known as cytosine arabinoside (Ara-C), was the very first marine-derived compound approved for its anticancer properties (Figure 3). It had been obtained from a Caibbean sponge, *Cryptotheca crypta*, synthesized for the first time in 1959 and then rendered by *Streptomyces griseus* fermentation. It belongs to the category of drugs known as antimetabolite and exerts its activity interfering with the DNA synthesis. Cytarabine is a pyrimidine analogue and differs from its natural counterpart (cytidine and deoxy cytidine) for the presence of sugar arabinose instead of ribose and deoxyribose. Once inside the cell, cytarabine is rapidly converted into the active triphosphate form, competing with cytidine to incorporate itself into DNA. The modified DNA structure and the inhibition of DNA polymerase caused by cytarabine, prevent DNA replication and repair (Barreca et al., 2020; Wu et al., 2021).

Cytarabine was approved by the FDA in 1969 for the treatment of acute myeloid leukaemia (AML). Later, other haematological malignancies such as lymphoblastic and myeloid leukaemia, both in acute and chronic phase. A liposomal formulation of the drug was developed, that improved the molecular stability and half-life of the drug and also allowed a prolonged exposure to tumour cells in the central nervous system (CNS). For this reason, primary CNS lymphomas are among the many off-label indications for cytarabine, and it has therefore also been tested for the palliative treatment of leptomeningeal carcinomatosis.

A phase III trial (DEPOSEIN) demonstrated that in BC patients with newly diagnosed leptomeningeal metastasis, the addition of intrathecal liposomal cytarabine to systemic treatment versus systemic treatment alone, prolonged disease related PFS. Leptomeningeal metastases PFS was 3.8 months in the combined arm versus 2.2 in the systemic treatment alone arm (HR 0.61, *p* = 0.04) in the intent-to-treat population (Le Rhun et al., 2020).

Despite its demonstrated activity, cytarabine holds severe side effects, with bone marrow suppression and pancytopenia being the most common ones; infection, musculoskeletal and connective tissue abnormalities arise in a smaller percentage of patients. When administered intrathecally, neurological complications can occur, ranging from a reversible self-limiting cerebellar syndrome, chemical meningitis, myelopathy, up to a more diffuse encephalopathy with seizures (Baker et al., 1991).

### 3.2 Minor groove binders

About 60 years ago, compounds extracted from the Caribbean Sea squirt *Ecteinascidia turbinata* were found to have a great activity in the inhibition of cell proliferation. Nonetheless, it took about three decades to isolate the bioactive molecule, ecteinascidin 743 (ET-743), that was synthetically produced for the first time only in 1996 (Cuevas and Francesch, 2009).

Trabectedin and its synthetized analogue lurbinectedin are two innovative anticancer alkaloids isolated from extracts of the Caribbean tunicate *Ecteinascidia Turbinate* (Figure 3). The two compounds are structurally and functionally related; they share the same pentacyclic skeleton and differ in the so-called ring C which confers specific pharmacokinetic and pharmacodynamic features (Allavena et al., 2022). For instance, lurbinectedin has a three-fold higher MTD and a four-fold lower volume of distribution than those of trabectedin, thus allowing higher dose-intensities without a meaningful increase in toxicities (Takahashi et al., 2016).

Lurbinectedin and trabectedin belong to the class of “minor groove binders” agents, in light of their cytotoxic effect depending on the interaction with the specific DNA site (Leal et al., 2010). Indeed, the two molecules bind covalently the central guanine of specific nucleotide triplets, mainly located close to promoters of protein-coding genes, inhibiting active transcription by the arrest and degradation of elongating RNA polymerase II (Nuñez et al., 2016). Subsequently, DNA repair systems and especially the transcription-coupled Nucleotide Excision Repair (tc-NER) recognize the lurbinectedin/trabectedin-DNA adduct and induced cell apoptosis, generating double-strand DNA breaks (DSBs). Therefore, the cytotoxic activity of lurbinectedin and trabectedin requires a functional intact NER mechanism while a further enhancement has been described in case of deficient Homologous Recombination Repair (HRR) pathway (Tavecchio et al., 2008). As a consequence, the lack of an efficient DNA repair process in these cells leads to increased unrepaired DSBs induced by the NER-drug complex, eventually resulting in lethal DNA damage and cell death (Soares et al., 2007). Moreover, *in vitro* and *in vivo* studies have shown that these agents can positively affect the tumour microenvironment by several mechanisms. First, they reduce the viability of tumour-associated macrophages (TAM) and induce their apoptotic cell death (Belgiovine et al., 2017), preventing the production of cytokines involved in cancer growth, downregulation of immune response and resistance to antineoplastic treatments. In addition to mitigation of the TAM-mediated immunosuppression, exposure to lurbinectedin/trabectedin-induced cell death seems to trigger an immune system response by increasing T cell infiltration and activation, questioning a possible synergistic role of this agent with immune checkpoint blockade (Xie et al., 2019).

#### 3.2.1 Trabectedin

Trabectidin an antitumoral drug discovered in 1969 obtained from a Caribbean squirt of the sea: the *Ecteinascidia turbinate.* Trabectedin was approved by FDA for the treatment of advanced soft tissue sarcomas (Demetri et al., 2016) and ovarian cancer (Monk et al., 2010).

Preclinical studies with trabectedin showed potent anticancer activity of the drug against cell lines of solid tumours, including BC, even at very low doses (1–10 ng/ml). However, the small number of BC patients in the 13 different phase I studies testing trabectedin in solid tumours and a very limited number of dedicated experiences challenged the role of trabectedin in ABC (Taamma et al., 2001; Takahashi et al., 2001; Dincalci and Zambelli, 2016). Of note, one of the rare phase I involving ABC patients was reported by Sessa et al. and investigated the safety profile and the anti-tumour activity of the combination of trabectedin and doxorubicin in advanced soft-tissue sarcoma (STS) and ABC, and reported some encouraging data (Sessa et al., 2009).

Trabectedin was also studied in several phase II trials conducted in patients with various forms of solid malignancies, and again the contribution of ABC patients was very limited. Indeed, according to a retrospective review of the role of trabectedin in 35 different phase II clinical trials, only 215/2,298 (9.3%) patients of ABC (Zelek et al., 2006).

A phase II randomized trial evaluating the safety and efficacy profile of trabectedin in ABC patients among different treatment schedules (every 3-weeks versus weekly regimen) showed a greater activity of trabectedin 1.3 mg/m2 administered once every 3 weeks, with a reasonable safety profile. Indeed, no relevant differences were observed as regard the most frequent drug-related AEs (transaminitis, nausea, and asthenia) except for neutropenia (40 vs. 15%); however, the higher ORR (12 vs. 3.7%) and PFS (3.1 vs. 2.0 months) in the 3-weeks arm made this dosing regimen the recommended one in ABC patients (Goldstein et al., 2014).

In terms of activity, trabectedin seems particularly attractive in BC with DNA damage repair defects and especially in BRCA1/2 mutated germline tumours and in the so-called sporadic “BRCAness” BC with specific somatic gene alterations (Peto et al., 1999). At 17%–20% of primary BC are thought to have one of these predictive genomic scars, the impact of the candidate molecular predictors would be substantial in such a prevalent neoplasm (Turner et al., 2004). Based on the previously described MoA, a more pronounced activity of trabectedin in BC harbouring HR deficiency has been postulated (García et al., 2013). Accordingly, Delaloge et al. investigated the efficacy and safety of trabectedin in BRCA1/2 mutant ABC. Of the 35 evaluable BRCA1/2 germline mutation carriers who participated in the trial, PR was documented in six patients (17%) with a median PFS of 3.9 months. Despite the limited sample size, this trial supported trabectedin monotherapy as an active and well-tolerated option in heavily pretreated ABC carrying germline BRCA1/2 mutation (Delaloge et al., 2014).

To further investigate the role of trabectedin in BRCA1 vs. BRCA 2 mutants ABC, a substudy analysis in 39 pretreated ABC suggested that ORR was higher in BRCA2-mutated patients than BRCA1-mutated patients (33.3 vs. 9.1%) with a longer disease stabilisation (25.0 vs. 9.1%) and longer median PFS (4.7 vs. 2.5 months) (Ghouadni et al., 2017).

In addition, a phase II trial explored the role of alternative HR-deficiency genes in the efficacy of trabectedin in ABC. Contrary to expectation, the expression of xeroderma pigmentosum gene (XPG) did not contribute to the prediction of the trabectedin response, raising the question of which genes the main role in the clinical trabectedin susceptibility in the context of HR deficiency (Awada et al., 2013).

#### 3.2.2 Lurbinectedin

Several phase I trials have investigated lurbinectedin activity in advanced solid tumours, including BC, alone or in combination with other drugs. Apart from some quite interesting results observed with the association of lurbinectedin and gemcitabine (one PR and five SD among six evaluable patients with ABC) (Paz-Ares et al., 2017), no particularly noteworthy clinical effects were found with other companion drugs, such as paclitaxel (Drilon et al., 2016) and capecitabine (NCT02210364).

As for trabectedin, the role of HR deficiency in strengthening lurbinectedin efficacy suggested a possible strong activity in BRCAness tumours. Accordingly, Cruz et al. (2018) performed a phase II trial investigating the activity of lurbinectedin in pre-treated germline BCRA1/2 mutant ABC. Patients were divided into two groups based on BRCA1/2 status: 54 patients with BRCA1/2 mutation vs. 34 with wild-type (WT) or unknown status. Lurbinectedin was administered at a flat dose of 7 mg (then modified to a dose of 3.5 mg/sqm) every 3 weeks. Among the BRCA mutated cohort, the primary endpoint of ORR was 41 vs. 9% in the WT cohort (crossing the futility border). As regards the safety profile, the most common toxicities were haematological (neutropenia, lymphopenia, and anaemia) and biochemical (AST, ALT and creatinine increased) abnormalities; most frequent non-laboratory AEs included fatigue and nausea, without differences between the two cohort of patients. The BSA-dose adjustment meaningfully reduced the overall incidence of grade 3 or 4 AEs. Translational analysis showed that resistance to lurbinectedin relied widely on alterations in NER-related genes. As previously reported for trabectedin (Ghouadni et al., 2017), an interesting higher benefit of lurbinectedin was found in BRCA2 vs. BRCA1 mutant ABC patients (ORR 61 vs. 26%, respectively), possibly due to the specific role of the BRCA2 protein in preventing the formation of RNA-DNA hybrids (R-loops) during the transcriptional process (Bhatia et al., 2014) with a more pronounced genomic instability under the pressure of the minor-groove binding drugs.

Apart from a completed but not yet published phase II trial (NCT02454972) regarding 21 germline BRCA1/2 mutant ABC, currently there are no ongoing trials of lurbinectedin in BC. Noteworthy, on June 2020, lurbinectedin received its first approval by the FDA for second-line treatment of patients with metastatic small-cell lung cancer (SCLC) progressing after platinum-containing chemotherapy, based on positive results of a single-arm, phase II basket trial (NCT02454972) (Trigo et al., 2020). However, a subsequent phase III trial (NCT02566993) comparing lurbinectedin plus doxorubicin with the common second-line treatments (topotecan or cyclophosphamide, doxorubicin plus vincristine regimen) eventually failed to show a significant OS benefit (Paz-Ares et al., 2021).

## 4 Antibody-drug conjugates

**FIGURE 4:**
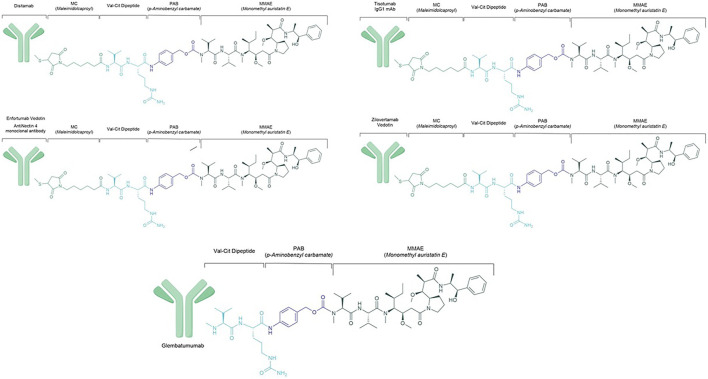
Chemical structure of main monomethyl auristatin E ADCs.

ADC are considered the latest achievement in the landscape of tailored cancer treatment. The mechanism of action of these drugs is to deliver a cytotoxic payload attached, *via* a cleavable linker, to an antibody that targets a specific surface antigen expressed by the cancer and its niche (Boni et al., 2020). This smart way of delivering chemotherapy is currently used in HER2-positive BC with the advent of trastuzumab-emtansine (T-DM1) (von Minckwitz et al., 2019), and trastuzumab deruxtecan (Cortés et al., 2022; Modi et al., 2022) and in TNBC with Sacituzumab govitecan (Bardia et al., 2021), having different linkers and payloads. Among these payloads, a marine compound used in several ADCs is the monomethyl auristatin E (MMAE), a potent cytotoxic agent derived from the *dolastatins*, pseudopeptides extracted from shell-less marine mollusc *Dolabela Auricularia* (Dosio et al., 2011) (Figure 4). Isolation dates back to 1987 when auristatins, synthetic analogues of the natural antimitotic agent *dolastatin 10,* were extracted from *Dolabella auricularia* by Pettit and colleagues (Bai et al., 1990). Its ability to inhibit microtubule polymerisation and tubulin-dependent GTP hydrolysis leads to cell death. Aside with its potency, significant toxicities have been observed at doses insufficient to achieve clinical efficacy (Kumar et al., 2017). The release of MMAE molecules in circulation leads to cell apoptosis, inhibition of cell growth and angiogenesis. The main AEs associated with MMAE payload are myelotoxicity (anaemia and neutropenia) and peripheral neuropathy. In addition to these AEs, antibody-dependent side effects must also be considered.

The linker that binds MMAE to the antibody is stable in the extracellular fluid, but is cleaved by cathepsin once ADC has bound to and entered the antigen of the target cancer cell, whereupon ADC releases the toxic MMAE and activates the potent antimitotic mechanism (Caculitan et al., 2017).

Most ADCs have a particular property called the “bystander effect.” After ADC binding, the cytotoxic molecules are released not only to cells expressing the target but also to adjacent or nearby cells. At the same time, this particular mechanism also damages stromal tumour cells and vascularisation, thus increasing the killing effect of cancer cells. Another important feature is the Drug-to-Antibody Ratio (DAR), defined as the number of payload molecules linked to each antibody, which is fundamental for determining the toxicity and the activity of the drug.

### 4.1 Enfortumab vedotin

Enfortumab vedotin (EV) targets cells expressing nectin-4. Nectin-4 is a member of the nectin family of immunoglobulin-like adhesion molecules mediating Ca^2+^-independent cell–cell adhesion processes (Rikitake et al., 2012). The immunohistochemistry analyses demonstrated a moderate to strong expression (H-score > 100) of nectin-4 in about 50% of BC specimens. AGS-22M6, a fully human antibody targeting nectin-4, was studied *in vitro* and *in vivo* for affinity, cross-reactivity and ability to induce cell apoptosis. AGS-22M6 conjugated to MMAE showed a dose-dependent activity *in vivo*, inhibiting cancer cell growth at low doses and inducing tumour regression at higher doses (Challita-Eid et al., 2016).

The EV-202 trial is an open-label phase II study (NCT04225117) evaluating ORR in previously treated locally advanced/metastatic malignant solid tumours, including HR-positive and HER2-negative BC and TNBC. All patients receive EV 1.25 mg/kg IV on Days 1,8, and 15 of each 28-days cycle until progression or unacceptable toxicity (Bruce et al., 2020). The study is currently ongoing and is expected to be completed in April 2024.

Noteworthy, in December 2019 EV obtained the FDA approval for locally advanced or metastatic urothelial cancer in previously pre-treated patients, based on the significant longer OS results observed with EV compared to standard chemotherapy (Powles et al., 2021).

### 4.2 Ladiratuzumab vedotin

Ladiratuzumab vedotin (LV) is an ADC whose IgG1 antibody targets the zinc transporter LIV-1, expressed in ER + BC cells, with a MMAE payload. A discrete tolerability profile and activity have been proven in pre-treated advanced TNBC patients at the recommended dose of 2.5 mg/kg every 21 days, with a disease control rate (DCR) up to 59%, mostly with SD (Modi et al., 2018). Recently presented data showed an ORR around 28% (95% CI: 13, 47) with a weekly schedule at 1.25 mg/kg (Tsai et al., 2021). Ongoing studies on safety and tolerability profile in metastatic BC are exploring LV alone or in combination with trastuzumab (NCT01969643). Other combinations regard immunotherapy with PDL1 inhibitors. An open-label phase Ib/II trial (SGNLVA-002/KEYNOTE 721) is currently investigating the activity of LV combined with pembrolizumab (200 mg every 3-weeks) in treatment-naïve (locally) advanced TNBC. The rationale lies behind the concept that LV might produce an advantageous microenvironment for the engagement of the immune-response enhanced by the anti-PD-1 drug. This combination showed encouraging data in 26 TNBC patients, with an ORR of 54% (95% CI: 33.4, 73.4) and a manageable toxicity profile comprising fatigue, alopecia, gastrointestinal symptoms, and peripheral neuropathy, mostly low grade. Further immunotherapy-based regimens are being investigated in an umbrella randomised trial which includes LV alone or in combination with atezolizumab (anti-PD-L1) in advanced TNBC (NCT03424005). The use of LV was also investigated in the neoadjuvant setting in the I-SPY2 trial (NCT01042379), resulting similar in pathological complete response (pCR) and AEs to paclitaxel, despite less incidence of CIPN(Beckwith et al., 2021).

### 4.3 Tisotumab vedotin

Tisotumab vedotin is an ADC directed toward tissue factor (TF) linked with MMAE. Monoclonal antibodies or pathway inhibitors directed to TF have been demonstrated to inhibit cancer cell growth, metastases spreading and angiogenesis. In preclinical studies, high levels of TF are expressed in invasive tumours, particularly in TNBC (Zhang et al., 2017). No other studies are currently recruiting patients with BC. However, based on this evidence, there is room for exploration of this drug, particularly in TNBC.

On 20 September 2021, tisotumab vedotin was approved by the FDA for the treatment of previously treated metastatic cervical cancer based on an ORR of 24% (95% CI: 16, 33) in the NCT03438396 phase II trial (Coleman et al., 2021).

### 4.4 Disitamab vedotin

Another effective ADC is disitamab vedotin (RC48-ADC), which consists of a HER2 monoclonal antibody bound to the MMAE payload with a DAR of 4 by a cleavable protease linker. Upon binding, the MMAE is released into lysosomes and produces a variety of compounds that are conjugated or non-conjugated to trastuzumab in varying proportions, enhancing the cytotoxic activity of both drugs with high affinity and specificity (Yao et al., 2015; Abdollahpour-Alitappeh et al., 2019). *In vitro* studies confirmed that conjugated trastuzumab is more effective than unconjugated trastuzumab in inhibiting colony formation in HER2-positive cells, making the RC48-ADC a potential therapeutic option in HER2-positive BC (Yaghoubi et al., 2021).

A pooled analysis of two phase I studies on RC48-ADC (NCT02881138 and NCT03052634) have shown increasing response in terms of tumour shrinkage and PFS at higher doses, achieving an ORR of 40.0% (95% CI: 21.1, 61.3) and PFS of 6.3 months (95% CI: 4.3, 8.8) with a dose of 2.5 mg/kg in pre-treated HER2-postive BC (Wang et al., 2018). Similar results were observed in the HER2-low subgroup of BC. Hepatic function alteration and neuropathy were reported in 3/4 of cases, and neutropenia in nearly half. Most treatment-related adverse events (TRAEs) were mild to moderate in severity. The most favourable profile in terms of benefit-risk ratio occurred with a fortnightly administration at a dose of 2.0 mg/kg (Wang et al., 2021). Ongoing studies in previously treated HER2-positive BC are currently recruiting patients to test the drug efficacy in phase II and III clinical trials (NCT03500380; NCT04400695) and in neoadjuvant settings (NCT05134519). Noteworthy, to date, disitamab vedotin has received its first approval in China for the treatment of HER2-positive advanced gastric cancer (Deeks, 2021).

### 4.5 ALT-P7

A phase I trial on ALT-P7, a trastuzumab conjugated with two MMAE molecules, enrolled patients with advanced HER2-positive BC previously treated with at least two anti-HER2 therapies. ALT-P7 was well tolerated up to a dose of 4.2 mg/kg with DLT observed at 4.8 mg/kg. In pilot experience, twenty-two patients had an assessment at 6 weeks with a disease control rate of 77.3% (17/22), and a partial response in 2/15 cases with measurable disease (Park et al., 2020).

### 4.6 Zilovertamab vedotin

The expression of receptor tyrosine kinase-like orphan receptors (ROR) activated by noncanonical Wnt signalling pathway could represent a potential target for ADC therapy in BC (Zhang et al., 2012). ROR1 is targeted by an ADC called zilovertamab vedotin (ZV) which showed a fast internalisation and effective MMAE release. Preliminary evidence of strong anticancer activity in terms of ORRs have been documented for lymphoma; studies in TNBC are still ongoing (NCT04504916).

### 4.7 Glembatumumab vedotin

Glembatumumab vedotin (GV) consists of an antibody directed against NMB glycoprotein (gpNMB), a negative prognostic marker overexpressed in cancer cells, conjugated to MMAE (Maric et al., 2013). The randomised phase II trial EMERGE demonstrated no significant differences in ORR as compare to standard chemotherapy (6 vs. 7%), but the activity of GV appeared to be increased in TNBC and especially in case of gpNMB-overexpression (≥25% epithelial cancer cells staining positive by IHC) (Yardley et al., 2015). In the METRIC trial, patients with pretreated advanced TNBC overexpressing gpNMB were selected to receive GV or capecitabine at randomisation. Interestingly, the GV showed greater activity in tumour shrinkage compared with capecitabine, but with a shorter and transient duration of response (Vahdat et al., 2021).

### 4.8 Lonigutamab Ugodotin

Lonigutamab Ugodotin (W0101) is an ADC which targets the Insulin-like Growth Factor 1 receptor (IGF-1R) and delivers MMEA as a payload. A phase I trial has shown that it is able to induce tumour regression in BC models with IGF-1R overexpression without affecting normal cells (Akla et al., 2020). A first-ever pilot clinical trial (NCT03316638) is currently evaluating the safety profile of the drug in advanced or metastatic tumours, including BC.

## 5 Conclusion

To date, the only marine-derived drug approved for BC treatment is eribulin. As discussed, several other agents have been (or are still being) evaluated in clinical trials for the treatment of BC. Even though these agents have not yet entered the phase III phase, we believe that interesting progress is being made in studying these drugs in BC as well (Table 1).

**TABLE 1:** Studies on marine-derived compounds in breast cancer. Abbreviations: TPC, treatment physician’s choice; BC, Breast Cancer; ABC, advanced breast cancer; LA, locally advanced; HR, Hormone Receptor; TNBC, triple-negative breast cancer; LM, leptomeningeal metastasis; OS, overall survival; ORR, objective response rate; PFS, progression free survival; DCR, disease control rate; pCR, pathological complete response; PR, partial response; SD, stable disease; DTL, Dose Limiting Toxicity; AE, Adverse Events; MTD, maximal tolerance dose; RP2D, recommended phase II dose; NA, not available.

Drug	Phase	Sample size (BC)	Main results/Primary endpoint	Authors/NCT number
Marine-drugs with activity in BC treatment - APPROVED				
Eribulin Mesylate	III	762	mOS = 13.1 vs 10.6 months (HR 0.81, *p* = 0.0041) mPFS = 3.7 vs 2.2 months (HR 0.87, *p* = 0.137) ORR = 12% vs 5% (*p* = 0.002)	Cortes et al. (2011)
	III	1102	mOS = 15.9 vs 14.5 months (HR 0.88, *p* = 0.056) mPFS = 4.1 vs 4.2 months (HR 1.08, *p* = 0.30) ORR = 11 vs 11.5% (*p* = 0.85)	Kaufman et al. (2015)
Intrathecal Liposomal cytarabine	III	74	mLM-PFS = 3.8 vs 2.2 months (HR 0.61, *p* = 0.04)	Le Rhun et al. (2020)
Marine-drugs with activity in BC treatment - NOT APPROVED				
Ladiratuzumab vedotin	I	44	ORR=32%	Modi et al. (2018)
	I	29	ORR = 28% (95% CI: 13, 47)	Tsai et al. (2021)
	I	26	ORR = 54% (95% CI: 33.4, 73.4)	Han et al. 2020
Disatamab vedotinI	I	70	Dose of 1.5 mg/kg: ORR = 22.2% (95% CI: 6.4, 47.6); mPFS = 4.0 months (95% CI: 2.6, 7.6)Dose of 2.0 mg/kg: ORR = 42.9% (95% CI: 21.8, 66.0); mPFS = 5.7 months (95% CI: 5.3, 8.4)Dose of 2.5 mg/kg: ORR = 40.0% (95% CI: 21.1, 61.3); mPFS = 6.3 months (95% CI: 4.3, 8.8)	Wang et al. (2018)
		48	ORR = 39.6% (95% CI: 25.8, 54.7)mPFS = 5.7 months (95% CI: 4.1, 8.3)	
ALT-P7	I	22	DCR at 6 weeks = 77.3%(17/22) PR = 13.3% (2/15)	Park et al. (2020)
Glembatumumab vedotin	II	83	ORR = 6% (5/83) for GV vs 7% (3/41) for ChemotherapyORR = 30% (7/23) vs 9% (1/11) for gpNMB overexpression (≥ 25% of tumor cells)	Yardley et al. (2015)
	IIB	213	mPFS = 2.9 months (95% CI: 2.8, 3.5) for the GV arm vs 2.8 months (95% CI: 1.6, 3.2) months for the capecitabine arm (HR = 0.95; 95% CI: 0.71, 1.29; p = 0.7607)	Vahdat et al. (2021)
Trabectedin	II	27	ORR = 14% (95% CI: 3.5–32%) mOS = 10 months (95% CI: 4.88–15.18 months)	Zelek et al. (2006)
	I	9	PR 55.5% (5/9), SD 33.3 (3/9)	Sessa et al (2009)
	II	44	mPFS = 1.9 months (95% CI: 1.8-3.5)PR 15.9% (7/44)	Awada et al, (2013)
	II	40	ORR = 17% (95% CI: 7,34)	Delaloge et al. (2014)
Lurbinectedin	II	54	ORR = 41% (95% CI: 28% to 55%)	Cruz et al. (2018)
		34	ORR = 9% (95% CI: 2% to 24%)	
	I	11	ORR = 17% (1/6); SD 67% (4/6)	Paz-Ares et al. (2017)
Plocabulin	I	5	SD 60% (3/5)	Elez et al. (2019)
	Marine-drugs with activity in BC treatment - ONGOING CLINICAL TRIALS				
RC48-ADC	II	20	pCR	NCT05134519
	III	366	PFS	NCT04400695
	Ib	112	RP2D	NCT03052634
	II/III	301	PFS	NCT03500380
CAB-ROR2-ADC (BA3021)	I/II	420	Safety Profile; AEs (Phase I)ORR	NCT03504488
			(Phase II)E	
Enfortumab Vedotin	II	280	ORR	NCT04225117
Ladiratuzumab Vedotin		4000	pCR	NCT01042379
	b/II	211	ORR; AEs; Incidence of laboratory abnormalities; DLT	NCT03310957
	Ib/II	280	ORR; AEs; Incidence of laboratory abnormalities; DLT	NCT01969643
W0101	I/II	316	AEs	NCT03316638
Zilovertamab vedotin	II	210	ORR	NCT04504916

Pharmacologic agents from natural products have always been used in the treatment of human diseases. The success rate for natural products to be developed into drugs is higher than in case of synthetic compounds (0.3 vs. 0.001%) (Atanasov et al., 2021). Among these, marine natural compounds show higher incidence of significant bioactivity which is associated with their rare and unique chemical structure. Indeed, they typically present both a direct and an indirect action on tumour cells and tumour microenvironment contrary to classical chemotherapy agents with a specific cytotoxic activity (i.e., alkylating agents, antimetabolites, topoisomerase II inhibitors). This is particularly evident for eribulin, plocabulin, trabectedin and lurbinectedin. Eribulin causes tumor cells apoptosis by microtubule-targeting mechanisms but it also acts on tumour microenvironment, angiogenesis and epithelial-mesenchymal transition. Plocabulin has both a peculiar microtubule dynamics inhibition and a powerful vascular-disrupting activity. The mechanism of action of trabectedin and lurbinectedin also involves a direct cytotoxic mechanism on cancer cells and a modulation of transcription regulation of cancer and normal cells (i.e., macrophages) thus leading to microenvironment changes. As a result of this mechanism of action, as known from the experience of trabectedin in soft tissue sarcomas, it seems likely that trabectedin antitumor activity is more frequently associated with a disease stabilization, even prolonged, but not necessarily with an objective response rate according to RECIST criteria (De Sanctis et al., 2016). Furthermore, ADCs act not only on cells expressing the target antigen but also on off-target cells and tumor microenvironment, throughout the bystander effect. Some pharmacological characteristics, such as the hydrophobicity of the marine-derived payload auristatin, seem to play a major role in this effect.

Therefore, the antitumor activity of these compounds seems to arise from a combination of more than one mechanism and this may explain their predominant use as single agents as opposed to classical chemotherapeutic agents (especially in the adjuvant and neoadjuvant settings, where cytotoxic agents with different mechanisms of action are used in order to inhibit the emergence of broad spectrum drug resistance).

Notably, there must be thousands or even millions of, as yet, undiscovered marine organisms that may provide interesting new anticancer agents in the future. However, developing a new drug from a natural product is challenging and requires an interdisciplinary approach. Indeed, this long-term process typically takes 20–30 years and includes basic research, preclinical and clinical trials, but we believe that it is worth to be funded. Furthermore, a better knowledge of the factors involved in the sensitivity of individual tumour (and tumour subtype) might lead to a more rational and effective use of newly discovered marine-derived drugs.

## Data Availability

No new data were created or analysed in this study. Data sharing is not applicable to this article.

## References

[B1] Abdollahpour-Alitappeh M. Lotfinia M. Bagheri N. Sineh Sepehr K. Habibi-Anbouhi M. Kobarfard F. (2019). Trastuzumab-monomethyl auristatin E conjugate exhibits potent cytotoxic activity *in vitro* against HER2-positive human breast cancer. J. Cell. Physiol. 234, 2693–2704. 10.1002/jcp.27085 30246298

[B2] Akla B. Broussas M. Loukili N. Robert A. Beau-Larvor C. Malissard M. (2020). Efficacy of the antibody-drug conjugate W0101 in preclinical models of IGF-1 receptor overexpressing solid tumors. Mol. Cancer Ther. 19, 168–177. 10.1158/1535-7163.MCT-19-0219 31594825

[B3] Allavena P. Belgiovine C. Digifico E. Frapolli R. D’Incalci M. (2022). Effects of the anti-tumor agents trabectedin and lurbinectedin on immune cells of the tumor microenvironment. Front. Oncol. 12, 851790. 10.3389/FONC.2022.851790 35299737 PMC8921639

[B4] Araki K. Fukada I. Yanagi H. Kobayashi K. Shibayama T. Horii R. (2017). First report of eribulin in combination with pertuzumab and trastuzumab for advanced HER2-positive breast cancer. Breast 35, 78–84. 10.1016/J.BREAST.2017.06.015 28662406

[B5] Atanasov A. G. Zotchev S. B. Dirsch V. M. Orhan I. E. Banach M. Rollinger J. M. (2021). Natural products in drug discovery: Advances and opportunities. Nat. Rev. Drug Discov. 20, 200–216. 10.1038/S41573-020-00114-Z 33510482 PMC7841765

[B6] Awada A. Cortes J. Martin M. Aftimos P. Oliveira M. Espie M. (2013). Final results of a phase II trial of trabectedin (T) in patients with hormone receptor-positive, HER2-negative advanced breast cancer, according to xeroderma pigmentosum gene (XPG) expression. J. Clin. Oncol. 31, 547. 10.1200/JCO.2013.31.15_SUPPL.547 27266804

[B7] Bai R. L. Pettit G. R. Hamel E. (1990). Structure-activity studies with chiral isomers and with segments of the antimitotic marine peptide dolastatin 10. Biochem. Pharmacol. 40, 1859–1864. 10.1016/0006-2952(90)90367-t 2242019

[B8] Baker W. J. Royer G. L. Weiss R. B. (1991). Cytarabine and neurologic toxicity. J. Clin. Oncol. 9, 679–693. 10.1200/JCO.1991.9.4.679 1648599

[B9] Bardia A. Hurvitz S. A. Tolaney S. M. Loirat D. Punie K. Oliveira M. (2021). Sacituzumab govitecan in metastatic triple-negative breast cancer. N. Engl. J. Med. 384, 1529–1541. 10.1056/NEJMoa2028485 33882206

[B10] Barreca M. Spanò V. Montalbano A. Cueto M. Díaz Marrero A. R. Deniz I. (2020). Marine anticancer agents: An overview with a particular focus on their chemical classes. Mar. Drugs 18, E619. 10.3390/MD18120619 33291602 PMC7761941

[B11] Beckwith H. Schwab R. Yau C. Stringer-Reasor E. Wei S. Chien A. J. (2021). Abstract PD1-10: Evaluation of SGN-LIV1a followed by AC in high-risk HER2 negative stage II/III breast cancer: Results from the I-SPY 2 TRIAL. Cancer Res. 81, PD1-10. 10.1158/1538-7445.SABCS20-PD1-10

[B12] Belgiovine C. Bello E. Liguori M. Craparotta I. Mannarino L. Paracchini L. (2017). Lurbinectedin reduces tumour-associated macrophages and the inflammatory tumour microenvironment in preclinical models. Br. J. Cancer 117, 628–638. 10.1038/BJC.2017.205 28683469 PMC5572168

[B13] Bhatia V. Barroso S. I. García-Rubio M. L. Tumini E. Herrera-Moyano E. Aguilera A. (2014). BRCA2 prevents R-loop accumulation and associates with TREX-2 mRNA export factor PCID2. Nature 511, 362–365. 10.1038/NATURE13374 24896180

[B14] Boni V. Sharma M. R. Patnaik A. (2020). The resurgence of antibody drug conjugates in cancer therapeutics: Novel targets and payloads. Am. Soc. Clin. Oncol. Educ. Book. 40, 1–17. 10.1200/EDBK_281107 32315240

[B15] Brönstrup M. Sasse F. (2020). Natural products targeting the elongation phase of eukaryotic protein biosynthesis. Nat. Prod. Rep. 37, 752–762. 10.1039/D0NP00011F 32428051

[B16] Bruce J. Y. Pusztai L. Braiteh F. S. Gorla S. R. Wu C. Baranda J. (2020). EV-202: A phase II study of enfortumab vedotin in patients with select previously treated locally advanced or metastatic solid tumors. J. Clin. Oncol. 38, TPS3647. 10.1200/JCO.2020.38.15_suppl.TPS3647

[B17] Caculitan N. G. Dela Cruz Chuh J. Ma Y. Zhang D. Kozak K. R. Liu Y. (2017). Cathepsin B is dispensable for cellular processing of cathepsin B-cleavable antibody-drug conjugates. Cancer Res. 77, 7027–7037. 10.1158/0008-5472.CAN-17-2391 29046337

[B18] Challita-Eid P. M. Satpayev D. Yang P. An Z. Morrison K. Shostak Y. (2016). Enfortumab vedotin antibody–drug conjugate targeting nectin-4 is a highly potent therapeutic agent in multiple preclinical cancer models. Cancer Res. 76, 3003–3013. 10.1158/0008-5472.CAN-15-1313 27013195

[B19] Coleman R. L. Lorusso D. Gennigens C. González-Martín A. Randall L. Cibula D. (2021). Efficacy and safety of tisotumab vedotin in previously treated recurrent or metastatic cervical cancer (innovaTV 204/GOG-3023/ENGOT-cx6): A multicentre, open-label, single-arm, phase 2 study. Lancet. Oncol. 22, 609–619. 10.1016/S1470-2045(21)00056-5 33845034

[B20] Cortés J. Kim S.-B. Chung W.-P. Im S.-A. Park Y. H. Hegg R. (2022). Trastuzumab deruxtecan versus trastuzumab emtansine for breast cancer. N. Engl. J. Med. 386, 1143–1154. 10.1056/NEJMoa2115022 35320644

[B21] Cortes J. O’Shaughnessy J. Loesch D. Blum J. L. Vahdat L. T. Petrakova K. (2011). Eribulin monotherapy versus treatment of physician’s choice in patients with metastatic breast cancer (EMBRACE): A phase 3 open-label randomised study. Lancet (London, Engl. 377, 914–923. 10.1016/S0140-6736(11)60070-6 21376385

[B22] Cruz C. Llop-Guevara A. Garber J. E. Arun B. K. Perez Fidalgo J. A. Lluch A. (2018). Multicenter phase II study of lurbinectedin in BRCA-mutated and unselected metastatic advanced breast cancer and biomarker assessment substudy. J. Clin. Oncol. 36, 3134–3143. 10.1200/JCO.2018.78.6558 30240327 PMC6209089

[B23] Cuevas C. Francesch A. (2009). Development of Yondelis (trabectedin, ET-743). A semisynthetic process solves the supply problem. Nat. Prod. Rep. 26, 322–337. 10.1039/b808331m 19240944

[B24] De Sanctis R. Agostinetto E. Masci G. Ferraro E. Losurdo A. Viganò A. (2018). Predictive factors of eribulin activity in metastatic breast cancer patients. Oncology 94 Suppl 1, 19–28. 10.1159/000489065 PMC6193748 30036884

[B25] De Sanctis R. Marrari A. Santoro A. (2016). Trabectedin for the treatment of soft tissue sarcomas. Expert Opin. Pharmacother. 17, 1569–1577. 10.1080/14656566.2016.1204295 27328277

[B26] Deeks E. D. (2021). Disitamab vedotin: First approval. Drugs 81, 1929–1935. 10.1007/S40265-021-01614-X 34661865

[B27] Delaloge S. Wolp-Diniz R. Byrski T. Blum J. L. Gonçalves A. Campone M. (2014). Activity of trabectedin in germline BRCA1/2-mutated metastatic breast cancer: Results of an international first-in-class phase II study. Ann. Oncol. 25, 1152–1158. 10.1093/ANNONC/MDU134 24692579

[B28] Demetri G. D. von Mehren M. Jones R. L. Hensley M. L. Schuetze S. M. Staddon A. (2016). Efficacy and safety of trabectedin or dacarbazine for metastatic liposarcoma or leiomyosarcoma after failure of conventional chemotherapy: Results of a phase III randomized multicenter clinical trial. J. Clin. Oncol. 34, 786–793. 10.1200/JCO.2015.62.4734 26371143 PMC5070559

[B29] Dincalci M. Zambelli A. (2016). Trabectedin for the treatment of breast cancer. Expert Opin. Investig. Drugs 25, 105–115. 10.1517/13543784.2016.1124086 26592307

[B30] Dosio F. Brusa P. Cattel L. (2011). Immunotoxins and anticancer drug conjugate assemblies: The role of the linkage between components. Toxins (Basel) 3, 848–883. 10.3390/toxins3070848 22069744 PMC3202854

[B31] Drilon A. Garralda E. Stathis A. Szyldergemajn S. Hyman D. Boni V. (2016). Lurbinectedin (PM01183) plus paclitaxel (P), recommended dose (RD) expansion results with or without the addition of bevacizumab (Bev) in patients (pts) with selected solid tumors. Ann. Oncol. 27, vi125. 10.1093/ANNONC/MDW368.34

[B32] Dyshlovoy S. A. Honecker F. (2018). Marine compounds and cancer: 2017 updates. Mar. Drugs 16, E41. 10.3390/MD16020041 29364147 PMC5852469

[B33] Dyshlovoy S. A. Honecker F. (2015). Marine compounds and cancer: Where do we stand? Mar. Drugs 13, 5657–5665. 10.3390/MD13095657 26540740 PMC4584346

[B34] Elez E. Gomez-Roca C. Soto Matos-Pita A. Argiles G. Valentin T. Coronado C. (2019). First-in-human phase I study of the microtubule inhibitor plocabulin in patients with advanced solid tumors. Invest. New Drugs 37, 674–683. 10.1007/S10637-018-0674-X 30411218

[B35] Galmarini C. M. Martin M. Bouchet B. P. Guillen-Navarro M. J. Martínez-Diez M. Martinez-Leal J. F. (2018). Plocabulin, a novel tubulin-binding agent, inhibits angiogenesis by modulation of microtubule dynamics in endothelial cells. BMC Cancer 18, 164. 10.1186/S12885-018-4086-2 29415678 PMC5803861

[B36] García M. J. Saucedo-Cuevas L. P. Muñoz-Repeto I. Fernández V. Robles M. J. Domingo S. (2013). Analysis of DNA repair-related genes in breast cancer reveals CUL4A ubiquitin ligase as a novel biomarker of trabectedin response. Mol. Cancer Ther. 12, 530–541. 10.1158/1535-7163.MCT-12-0768DNA-REPAIR-RELATED-GENES-IN-BREAST 23364677

[B37] Ghouadni A. Delaloge S. Lardelli P. Kahatt C. Byrski T. Blum J. L. (2017). Higher antitumor activity of trabectedin in germline BRCA2 carriers with advanced breast cancer as compared to BRCA1 carriers: A subset analysis of a dedicated phase II trial. Breast 34, 18–23. 10.1016/J.BREAST.2017.04.006 28467918

[B38] Goel S. Mita A. C. Mita M. Rowinsky E. K. Chu Q. S. Wong N. (2009). A phase I study of eribulin mesylate (E7389), a mechanistically novel inhibitor of microtubule dynamics, in patients with advanced solid malignancies. Clin. Cancer Res. 15, 4207–4212. 10.1158/1078-0432.CCR-08-2429 19509177

[B39] Goldstein L. J. Gurtler J. Del Prete S. A. Tjulandin S. Semiglazov V. F. Bayever E. (2014). Trabectedin as a single-agent treatment of advanced breast cancer after anthracycline and taxane treatment: A multicenter, randomized, phase II study comparing 2 administration regimens. Clin. Breast Cancer 14, 396–404. 10.1016/J.CLBC.2014.06.006 25239225

[B40] Gourmelon C. Frenel J. S. Campone M. (2011). Eribulin mesylate for the treatment of late-stage breast cancer. Expert Opin. Pharmacother. 12, 2883–2890. 10.1517/14656566.2011.637490 22087618

[B41] Ha M. W. Song B. R. Chung H. J. Paek S. M. (2019). Design and synthesis of anti-cancer chimera molecules based on marine natural products. Mar. Drugs 17, E500. 10.3390/MD17090500 31461968 PMC6780274

[B42] Kaklamani V. G. Jeruss J. S. Hughes E. Siziopikou K. Timms K. M. Gutin A. (2015). Phase II neoadjuvant clinical trial of carboplatin and eribulin in women with triple negative early-stage breast cancer (NCT01372579). Breast Cancer Res. Treat. 151, 629–638. 10.1007/S10549-015-3435-Y 26006067

[B43] Kashiwagi S. Asano Y. Goto W. Takada K. Takahashi K. Noda S. (2017). Use of Tumor-infiltrating lymphocytes (TILs) to predict the treatment response to eribulin chemotherapy in breast cancer. PLoS One 12, e0170634. 10.1371/JOURNAL.PONE.0170634 28166544 PMC5293550

[B44] Kaufman P. A. Awada A. Twelves C. Yelle L. Perez E. A. Velikova G. (2015). Phase III open-label randomized study of eribulin mesylate versus capecitabine in patients with locally advanced or metastatic breast cancer previously treated with an anthracycline and a taxane. J. Clin. Oncol. 33, 594–601. 10.1200/JCO.2013.52.4892 25605862 PMC4463422

[B45] Kumar A. White J. James Christie R. Dimasi N. Gao C. (2017). “Chapter twelve - antibody-drug conjugates,” Annu. Rep. Med. Chem. 50, 441–480. 10.1016/bs.armc.2017.08.002

[B46] Le Rhun E. Wallet J. Mailliez A. Le Deley M. C. Rodrigues I. Boulanger T. (2020). Intrathecal liposomal cytarabine plus systemic therapy versus systemic chemotherapy alone for newly diagnosed leptomeningeal metastasis from breast cancer. Neuro. Oncol. 22, 524–538. 10.1093/NEUONC/NOZ201 31637444 PMC7158648

[B47] Leal J. F. M. Martínez-Díez M. García-Hernández V. Moneo V. Domingo A. Bueren-Calabuig J. A. (2010). PM01183, a new DNA minor groove covalent binder with potent *in vitro* and *in vivo* anti-tumour activity. Br. J. Pharmacol. 161, 1099–1110. 10.1111/J.1476-5381.2010.00945.X 20977459 PMC2998690

[B48] Maric G. Rose A. A. Annis M. G. Siegel P. M. (2013). Glycoprotein non-metastatic b (gpnmb): A metastatic mediator and emerging therapeutic target in cancer. Onco. Targets. Ther. 6, 839–852. 10.2147/OTT.S44906 23874106 PMC3711880

[B49] Martínez-Díez M. Guillén-Navarro M. J. Pera B. Bouchet B. P. Martínez-Leal J. F. Barasoain I. (2014). PM060184, a new tubulin binding agent with potent antitumor activity including P-glycoprotein over-expressing tumors. Biochem. Pharmacol. 88, 291–302. 10.1016/J.BCP.2014.01.026 24486569

[B50] Modi S. Jacot W. Yamashita T. Sohn J. Vidal M. Tokunaga E. (2022). Trastuzumab deruxtecan in previously treated HER2-low advanced breast cancer. N. Engl. J. Med. 387, 9–20. 10.1056/NEJMoa2203690/NEJMOA2203690_DATA-SHARING 35665782 PMC10561652

[B51] Modi S. Pusztai L. Forero A. Mita M. Miller K. D. Weise A. (2018). Abstract PD3-14: Phase 1 study of the antibody-drug conjugate SGN-LIV1A in patients with heavily pretreated triple-negative metastatic breast cancer. Cancer Res. 78, PD3-14–14. 10.1158/1538-7445.SABCS17-PD3-14

[B52] Monk B. J. Herzog T. J. Kaye S. B. Krasner C. N. Vermorken J. B. Muggia F. M. (2010). Trabectedin plus pegylated liposomal Doxorubicin in recurrent ovarian cancer. J. Clin. Oncol. 28, 3107–3114. 10.1200/JCO.2009.25.4037 20516432

[B53] Nuñez G. S. Robles C. M. G. Giraudon C. Martínez-Leal J. F. Compe E. Coin F. (2016). Lurbinectedin specifically triggers the degradation of phosphorylated RNA polymerase II and the formation of DNA breaks in cancer cells. Mol. Cancer Ther. 15, 2399–2412. 10.1158/1535-7163.MCT-16-0172 27630271

[B54] Park Y. H. Ahn H. K. Kim J.-Y. Ahn J. S. Im Y.-H. Kim S.-H. (2020). First-in-human phase I study of ALT-P7, a HER2-targeting antibody-drug conjugate in patients with HER2-positive advanced breast cancer. J. Clin. Oncol. 38, 3551. 10.1200/JCO.2020.38.15_suppl.3551

[B55] Paz-Ares L. Ciuleanu T. Navarro A. Fulop A. Cousin S. Bonanno L. (2021). PL02.03 lurbinectedin/doxorubicin versus CAV or topotecan in relapsed SCLC patients: Phase III randomized ATLANTIS trial. J. Thorac. Oncol. 16, S844–S845. 10.1016/J.JTHO.2021.08.030

[B56] Paz-Ares L. Forster M. Boni V. Szyldergemajn S. Corral J. Turnbull S. (2017). Phase I clinical and pharmacokinetic study of PM01183 (a tetrahydroisoquinoline, Lurbinectedin) in combination with gemcitabine in patients with advanced solid tumors. Invest. New Drugs 35, 198–206. 10.1007/S10637-016-0410-3 27873130

[B57] Pedersini R. Vassalli L. Claps M. Tulla A. Rodella F. Grisanti S. (2018). Eribulin in heavily pretreated metastatic breast cancer patients in the real world: A retrospective study. Oncology 94 Suppl 1, 10–15. 10.1159/000489063 30036867 PMC6193751

[B58] Pera B. Barasoain I. Pantazopoulou A. Canales A. Matesanz R. Rodriguez-Salarichs J. (2013). New interfacial microtubule inhibitors of marine origin, PM050489/PM060184, with potent antitumor activity and a distinct mechanism. ACS Chem. Biol. 8, 2084–2094. 10.1021/CB400461J 23859655

[B59] Peto J. Collins N. Barfoot R. Seal S. Warren W. Rahman N. (1999). Prevalence of BRCA1 and BRCA2 gene mutations in patients with early-onset breast cancer. J. Natl. Cancer Inst. 91, 943–949. 10.1093/JNCI/91.11.943 10359546

[B60] Powles T. Rosenberg J. E. Sonpavde G. P. Loriot Y. Durán I. Lee J.-L. (2021). Enfortumab vedotin in previously treated advanced urothelial carcinoma. N. Engl. J. Med. 384, 1125–1135. 10.1056/NEJMoa2035807 33577729 PMC8450892

[B61] Rikitake Y. Mandai K. Takai Y. (2012). The role of nectins in different types of cell-cell adhesion. J. Cell. Sci. 125, 3713–3722. 10.1242/jcs.099572 23027581

[B62] Sakaguchi K. Nakatsukasa K. Koyama H. Kato M. Sakuyama A. Matsuda T. (2018). Phase II clinical trial of first-line eribulin plus trastuzumab for advanced or recurrent HER2-positive breast cancer. Anticancer Res. 38, 4073–4081. 10.21873/ANTICANRES.12697 29970533

[B63] Sessa C. Cresta S. Noberasco C. Capri G. Gallerani E. Braud F. D. (2009). Phase I clinical and pharmacokinetic study of trabectedin and cisplatin in solid tumours. Eur. J. Cancer 45, 2116–2122. 10.1016/J.EJCA.2009.04.002 19419856

[B64] Smith J. W. Vukelja S. Hoffman A. D. Jones V. E. McIntyre K. Berrak E. (2016). Phase II, multicenter, single-arm, feasibility study of eribulin combined with capecitabine for adjuvant treatment in estrogen receptor-positive, early-stage breast cancer. Clin. Breast Cancer 16, 31–37. 10.1016/J.CLBC.2015.07.007 26433876

[B65] Soares D. G. Escargueil A. E. Poindessous V. Sarasin A. De Gramont A. Bonatto D. (2007). Replication and homologous recombination repair regulate DNA double-strand break formation by the antitumor alkylator ecteinascidin 743. Proc. Natl. Acad. Sci. U. S. A. 104, 13062–13067. 10.1073/PNAS.0609877104 17656556 PMC1941813

[B66] Stonik V. A. (2009). Marine natural products: A way to new drugs. Acta Naturae 1, 15–25. 10.32607/20758251-2009-1-2-15-25 22649599 PMC3347521

[B67] Taamma A. Misset J. L. Riofrio M. Guzman C. Brain E. Lopez Lazaro L. (2001). Phase I and pharmacokinetic study of ecteinascidin-743, a new marine compound, administered as a 24-hour continuous infusion in patients with solid tumors. J. Clin. Oncol. 19, 1256–1265. 10.1200/JCO.2001.19.5.1256 11230466

[B68] Takahashi N. Li W. W. Banerjee D. Scotto K. W. Bertino J. R. (2001). Sequence-dependent enhancement of cytotoxicity produced by ecteinascidin 743 (ET-743) with doxorubicin or paclitaxel in soft tissue sarcoma cells. Clin. Cancer Res. 7, 3251–3257. 11595721

[B69] Takahashi R. Mabuchi S. Kawano M. Sasano T. Matsumoto Y. Kuroda H. (2016). Preclinical investigations of PM01183 (lurbinectedin) as a single agent or in combination with other anticancer agents for clear cell carcinoma of the ovary. PLoS One 11, e0151050. 10.1371/JOURNAL.PONE.0151050 26986199 PMC4795692

[B70] Tavecchio M. Simone M. Erba E. Chiolo I. Liberi G. Foiani M. (2008). Role of homologous recombination in trabectedin-induced DNA damage. Eur. J. Cancer 44, 609–618. 10.1016/J.EJCA.2008.01.003 18243687

[B71] Tolaney S. M. Kalinsky K. Kaklamani V. G. D’Adamo D. R. Aktan G. Tsai M. L. (2021). Eribulin plus pembrolizumab in patients with metastatic triple-negative breast cancer (ENHANCE 1): A phase Ib/II study. Clin. Cancer Res. 27, 3061–3068. 10.1158/1078-0432.CCR-20-4726 33727258

[B72] Trigo J. Subbiah V. Besse B. Moreno V. López R. Sala M. A. (2020). Lurbinectedin as second-line treatment for patients with small-cell lung cancer: A single-arm, open-label, phase 2 basket trial. Lancet. Oncol. 21, 645–654. 10.1016/S1470-2045(20)30068-1 32224306

[B73] Tsai M. Han H. S. Montero A. J. Tkaczuk K. H. Assad H. Pusztai L. (2021). 259P Weekly ladiratuzumab vedotin monotherapy for metastatic triple-negative breast cancer. Ann. Oncol. 32, S474–S475. 10.1016/j.annonc.2021.08.542

[B74] Turner N. Tutt A. Ashworth A. (2004). Hallmarks of “BRCAness” in sporadic cancers. Nat. Rev. Cancer 4, 814–819. 10.1038/NRC1457 15510162

[B75] Twelves C. Cortes J. Vahdat L. Olivo M. He Y. Kaufman P. A. (2014). Efficacy of eribulin in women with metastatic breast cancer: A pooled analysis of two phase 3 studies. Breast Cancer Res. Treat. 148, 553–561. 10.1007/S10549-014-3144-Y 25381136 PMC4243003

[B76] Ueda S. Saeki T. Takeuchi H. Shigekawa T. Yamane T. Kuji I. (2016). *In vivo* imaging of eribulin-induced reoxygenation in advanced breast cancer patients: A comparison to bevacizumab. Br. J. Cancer 114, 1212–1218. 10.1038/BJC.2016.122 27140309 PMC4891505

[B77] Vahdat L. T. Schmid P. Forero-Torres A. Blackwell K. Telli M. L. Melisko M. (2021). Glembatumumab vedotin for patients with metastatic, gpNMB overexpressing, triple-negative breast cancer (“METRIC”): A randomized multicenter study. npj Breast Cancer 7, 57. 10.1038/s41523-021-00244-6 34016993 PMC8137923

[B78] von Minckwitz G. Huang C.-S. Mano M. S. Loibl S. Mamounas E. P. Untch M. (2019). Trastuzumab emtansine for residual invasive HER2-positive breast cancer. N. Engl. J. Med. 380, 617–628. 10.1056/NEJMoa1814017 30516102

[B79] Wang J. Liu Y. Zhang Q. Feng J. Fang J. Chen X. (2021). RC48-ADC, a HER2-targeting antibody-drug conjugate, in patients with HER2-positive and HER2-low expressing advanced or metastatic breast cancer: A pooled analysis of two studies. J. Clin. Oncol. 39, 1022. 10.1200/JCO.2021.39.15_suppl.1022

[B80] Wang J. Xu B. Wang W. Fang J. (2018). An open-label, dose-escalation phase I study to evaluate RC48-ADC, a novel antibody-drug conjugate, in patients with HER2-positive metastatic breast cancer. J. Clin. Oncol. 36, 1030. 10.1200/JCO.2018.36.15_suppl.1030

[B81] Wu L. Ye K. Jiang S. Zhou G. (2021). Marine power on cancer: Drugs, lead compounds, and mechanisms. Mar. Drugs 19, 488. 10.3390/MD19090488 34564150 PMC8472172

[B82] Xie W. Forveille S. Iribarren K. Sauvat A. Senovilla L. Wang Y. (2019). Lurbinectedin synergizes with immune checkpoint blockade to generate anticancer immunity. Oncoimmunology 8, e1656502. 10.1080/2162402X.2019.1656502 31646106 PMC6791417

[B83] Yaghoubi S. Gharibi T. Karimi M. H. Sadeqi Nezhad M. Seifalian A. Tavakkol R. (2021). Development and biological assessment of MMAE-trastuzumab antibody-drug conjugates (ADCs). Breast Cancer 28, 216–225. 10.1007/s12282-020-01153-5 32889587

[B84] Yao X. Jiang J. Wang X. Huang C. Li D. Xie K. (2015). A novel humanized anti-HER2 antibody conjugated with MMAE exerts potent anti-tumor activity. Breast Cancer Res. Treat. 153, 123–133. 10.1007/s10549-015-3503-3 26253944

[B100] Yardley D. A. Weaver R. Melisko M. E. Saleh M. N. Arena F. P. Forero A. (2015). EMERGE: A Randomized Phase II Study of the Antibody-Drug Conjugate Glembatumumab Vedotin in Advanced Glycoprotein NMB-Expressing Breast Cancer. J. Clin. Oncol. 33 (14), 1609–1619. 10.1200/JCO.2014.56.2959 25847941

[B85] Zelek L. Yovine A. Brain E. Turpin F. Taamma A. Riofrio M. (2006). A phase II study of Yondelis (trabectedin, ET-743) as a 24-h continuous intravenous infusion in pretreated advanced breast cancer. Br. J. Cancer 94, 1610–1614. 10.1038/SJ.BJC.6603142 16736024 PMC2361304

[B86] Zhang S. Chen L. Cui B. Chuang H.-Y. Yu J. Wang-Rodriguez J. (2012). ROR1 is expressed in human breast cancer and associated with enhanced tumor-cell growth. PLoS One 7, e31127. 10.1371/journal.pone.0031127 22403610 PMC3293865

[B87] Zhang X. Li Q. Zhao H. Ma L. Meng T. Qian J. (2017). Pathological expression of tissue factor confers promising antitumor response to a novel therapeutic antibody SC1 in triple negative breast cancer and pancreatic adenocarcinoma. Oncotarget 8, 59086–59102. 10.18632/oncotarget.19175 28938620 PMC5601716

